# Space and Memory (Far) Beyond the Hippocampus: Many Subcortical Structures Also Support Cognitive Mapping and Mnemonic Processing

**DOI:** 10.3389/fncir.2019.00052

**Published:** 2019-08-07

**Authors:** Shane M. O’Mara, John P. Aggleton

**Affiliations:** ^1^School of Psychology and Institute of Neuroscience, Trinity College, Dublin, Ireland; ^2^School of Psychology, Cardiff University, Cardiff, United Kingdom

**Keywords:** memory, hippocampal formation, space, anterior thalamus, claustrum, diencephalic amnesia

## Abstract

Memory research remains focused on just a few brain structures—in particular, the hippocampal formation (the hippocampus and entorhinal cortex). Three key discoveries promote this continued focus: the striking demonstrations of enduring anterograde amnesia after bilateral hippocampal damage; the realization that synapses in the hippocampal formation are plastic e.g., when responding to short bursts of patterned stimulation (“long-term potentiation” or LTP); and the discovery of a panoply of spatially-tuned cells, principally surveyed in the hippocampal formation (place cells coding for position; head-direction cells, providing compass-like information; and grid cells, providing a metric for 3D space). Recent anatomical, behavioral, and electrophysiological work extends this picture to a growing network of subcortical brain structures, including the anterior thalamic nuclei, rostral midline thalamic nuclei, and the claustrum. There are, for example, spatially-tuned cells in all of these regions, including cells with properties similar to place cells of the hippocampus proper. These findings add new perspectives to what had been originally been proposed—but often overlooked—half a century ago: that damage to an extended network of structures connected to the hippocampal formation results in diencephalic amnesia. We suggest these new findings extend spatial signaling in the brain far beyond the hippocampal formation, with profound implications for theories of the neural bases of spatial and mnemonic functions.

## Introduction

An intensive research effort over the past four decades has revealed that discrete populations of cells, present in widely distributed networks in the brain, code for differing aspects of three-dimensional space (for comprehensive reviews, see Grieves and Jeffery, 2017; Hunsaker and Kesner, 2018). There has been a sustained research emphasis on the precise roles played by two connected structures (the entorhinal cortex and the hippocampus; collectively, the hippocampal formation) because these structures have been identified as having vital importance for certain types of memory (and, particularly, for spatial memory). Less attention has been given to the contributions from other structures connected to the hippocampal formation, although these structures seemingly play similarly vital roles in memory.

Here, we will focus on spatial mapping functions subserved by particular brain regions and networks, similar to those performed by the hippocampal formation: namely, the rostral midline and anterior nuclei of the thalamus, as well as the claustrum. These regions are less investigated and less well-understood than the hippocampal formation; they are not as frequently incorporated into theoretical accounts of spatial coding in the brain and are less well-recognized as spatial processing nodes (The one possible exception concerns the appreciation that the anterodorsal thalamic nucleus contains head-direction cells; Taube, 1995, 2007). Unexpectedly, there are cells in all of these subcortical sites that code for aspects of three-dimensional space, including positional information, boundary or perimeter information, as well as head directional and object information (Jankowski et al., 2014, 2015, 2017; Jankowski and O’Mara, 2015; Matulewicz et al., 2019). Moreover, it is now clear that lesions of the rostral and anterior thalamic nuclei can result in deficits in the performance of spatial and non-spatial mnemonic tasks that appear comparable to those resulting from lesions within the entorhinal-hippocampal axis. The few lesion investigations of the claustrum indicate that claustral lesions also disrupt certain aspects of spatial processing.

For clarity, we should note that the term “hippocampus” refers to the CA fields, dentate gyrus, and subiculum. Meanwhile the “hippocampal formation,” additionally includes the entorhinal cortex, presubiculum, and parasubiculum, which are also parts of the parahippocampal region (Witter, 2002). Some authorities treat the postsubiculum as distinct from the presubiculum (e.g., van Groen and Wyss, 1990b), and we shall follow this practice. Finally, the term “hippocampus proper” refers to the CA fields but not the subiculum.

## Memory Research Has Focused Especially on the Hippocampal Formation

The effort to understand how the brain encodes and supports memory functions has been underway for well over a century (Squire, 1987; Aggleton and Morris, 2018). Over that time, substantial progress has been made in elucidating the brain structures that support memory (including spatial memory), with one particular region remaining in the spotlight, namely, the hippocampal formation (Buzsáki and Moser, 2013). Memory research has focused particularly on the hippocampal formation for at least three interrelated reasons. The early description of the amnestic Patient HM demonstrated a clear and striking case of amnesia that has often been attributed to bilateral loss of the hippocampal formation (Scoville and Milner, 1957). Despite the fact that the surgery clearly involved additional gray and white matter (Annese et al., 2014), HM’s amnesia case-study continues to exercise a powerful hold on theoretical and experimental analyses in the memory literature. His near life-long, enduring and non-resolving amnesia, has provided, and continues to provide, a source of fertile hypotheses regarding the neural bases of learning and memory. A second reason for focusing on the hippocampal formation was the important and influential demonstration that synapses in the hippocampus are plastic, e.g., they display “long-term potentiation” (LTP), which results from a brief period (~1 s) of high-frequency electrical stimulation. While LTP was initially demonstrated by Bliss and Lømo (1973), it followed an original prediction by Hebb (1949). Subsequent demonstrations that pharmacological inhibitors of hippocampal LTP effectively inhibited learning and memory in spatial memory tasks, have also provided a rich source of theory and experiment focused on the role of the hippocampal formation in learning and memory (e.g., Bliss and Collingridge, 1993; van Praag et al., 1999; Lynch, 2004; Malenka and Bear, 2004; Larkin et al., 2008; Tsokas et al., 2016; Grewe et al., 2017). Added information has come from recent “engram” studies, which appear to show that the stimulation of specific hippocampal cell ensembles can reactivate a representation, e.g., a particular context (Tonegawa et al., 2015; Park et al., 2016).

The third powerful impetus derives from the work of John O’Keefe and his collaborators over the past four decades (e.g., O’Keefe and Dostrovsky, 1971; O’Keefe and Nadel, 1978, 1979; O’Keefe and Burgess, 1996; Krupic et al., 2015). In particular, O’Keefe’s work reanimated earlier suggestions by Tolman (1948) that there must be a “cognitive map” within the brain. Tolman’s experiments showed that rats were capable of engaging in “latent” learning—learning “on the fly” whilst exploring mazes, and subsequently using this information to solve route-finding problems in these self-same mazes when usual routes to reward were blocked (see also O’Mara, 2017). Tolman speculated that, contrary to the motor movement reinforcement theories of Clark Hull and others (e.g., Hull, 1943; Spence, 1956), rats were inducing or inferring something like a “survey map” of their environment, which allowed them to flexibly navigate that environment. O’Keefe’s demonstration of the existence of place cells in the hippocampus (O’Keefe and Dostrovsky, 1971) and his subsequent elaboration of the cognitive mapping theory with O’Keefe and Nadel (1978) in their book “*The Hippocampus as a Cognitive Map*” ensured that the hippocampus would remain a focus for investigations of spatial processing by the brain.

In the decades after O’Keefe’s initial demonstration of the existence of place cells, further discoveries indicated that the brain coded for differing aspects of three-dimensional space. In particular, the head-direction cells described by Taube et al. (1990a,b) suggested that, in addition to representations of place, the brain also maintains a representation of a compass-like heading in relation to the external three-dimensional world. However, missing from this picture was a sense of how the brain might code a metric for space—a sense of relative distance, and how a metric for space might play a role in spatial processing. The discovery of “grid cells” within the entorhinal cortex by Hafting et al. (2005) provided this missing link, powerfully reinforcing the idea that the entorhinal-hippocampal axis plays the central role in how the brain codes for space.

Current theories (e.g., Buzsáki and Moser, 2013) of the means by which the brain represents space (and memory) rely on an anatomical model revolving around the existence of cells representing position (place cells), heading (head-direction cells) and relative distance (grid cells). The key structures implicated are the hippocampus (principally concerned with representing spatial position), the postsubiculum and presubiculum, along with related anterior thalamic areas (principally concerned with representing heading information), and the entorhinal cortex (principally concerned with representing metric information). This tripartite anatomical-structural model now dominates theoretical views of spatial navigation and spatial representation. Biologically-inspired models of spatial navigation rely similarly on core ideas revolving around the dissociation of place information from heading information and their subsequent integration in other downstream areas to facilitate goal-directed navigation (e.g., Barrera and Weitzenfeld, 2008). We suggest that there are important components of this overall theoretical picture missing, in particular relating to evolutionarily-conserved brain regions that remain under-explored to this point. We further suggest that significant revisions of current models of how the brain controls behavioral choices in a spatial context are warranted.

**Figure 1:**
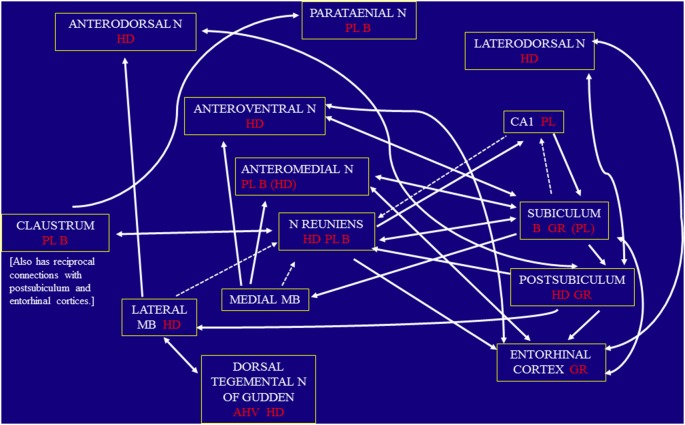
Schematic diagram showing the pattern of direct connections between the three key regions under consideration, namely the rostral thalamus, claustrum, and hippocampal formation. The dashed arrows indicate those connections that are especially light. The diagram also indicates the location of angular head velocity (AHV), border/perimeter (B), grid (GR), head-direction (HD), and place (PL) cells, based on current evidence. Parentheses indicate when the frequency of these spatial cells is low. Other abbreviations: MB, mammillary bodies; N, nucleus.

Here, we briefly review evidence suggesting that this dominant picture needs a considerable degree of extension. The hippocampal formation has vital interconnections with a wide variety of brain regions, so much so, in fact, that Aggleton and Brown (1999) suggested we should more properly think of an “extended hippocampal formation” when considering memory (see also Delay and Brion, 1969; Ranganath and Ritchey, 2012). Key elements within the extended hippocampal formation include the fornix, the mammillary bodies, the anterior thalamic nuclei, and retrosplenial cortex (Bubb et al., 2017). What are now archaic neuroanatomical constructions (such as “the trisynaptic loop”) are giving way to recent neuroanatomical and neurophysiological investigations, suggesting that interactions between the hippocampal formation, as a key spatial hub, in association with a distributed network of other key locations is a more appropriate functional neuroanatomical conception of the structures that support cognitive mapping (Figure 1).

## Evidence From Neodecorticate Animal Preparations

In the 1970s and 1980s a number of investigators investigated what functions could continue to be performed in the absence of a cerebral cortex and hippocampal formation. These preparations usually involved a bilateral removal of the developing cerebral cortex and hippocampus (decortication) at various time points postnatally. Such studies are rarely performed now. However, these early studies indicated that in the absence of a cortex and hippocampus, decorticate rats were capable of learning a variety of spatial and non-spatial tasks, suggesting that subcortical circuitry could, under some conditions, support processes such as nesting, food retrieval and foraging, and successfully returning to a point of origin, and the learning of complex spatial tasks where errors were prevented (for reviews, see Kolb and Whishaw, 1989; Whishaw, 1990). Furthermore, decorticate animals were also capable of successfully learning stimulus relations and responses in a variety of classical conditioning tasks, suggesting that the learning of classically-conditioned avoidance responses dependent on temporal relations remains intact in these animals. Subsequent investigations, particularly those using the rabbit classically-conditioned nictitating membrane preparation, have suggested that such learning requires intact cerebellar and brainstem nuclei (McCormick and Thompson, 1984; Thompson, 1986). In an interesting convergence, Rochefort et al. (2011) found that genetically-modified L7PKCI mice with deficits in PKC-dependent plasticity at parallel fiber–Purkinje cell synapses in the cerebellum have defective place cell processing in tasks requiring vestibular self-motion cues. Recent evidence (Watson et al., 2019) suggests there is a topographically-organized and direct anatomical cerebellar-hippocampal CA1 pathway; moreover, simultaneous local-field potential recordings conducted in the freely-moving animal disclosed synchronization of activity between area CA1 and cerebellum over behaviorally-relevant, subsecond, timescales during home-cage exploration and pellet retrieval on a linear track.

The key message from the early decortication studies is that there are subcortical circuits present capable of supporting complex chains of learned behavior in the absences of a cerebral cortex and hippocampus. The remaining subcortical structures include thalamic, brainstem, cerebellar and spinal structures, thus providing useful clues regarding the structures that might interact with hippocampal and cortical structures to support spatial and other forms of memory. Subsequent electrophysiological, anatomical and transgenic data suggest that there is a profound modulation of hippocampal processing by motor state. It will be of great interest in future experiments to focus on the interactions between the hippocampal formation and subcortical structures to understand the crucial contributions of these structures during spatial and mnemonic processing.

## Event Memory and the Anterior Thalamic Nuclei

More than 80 years ago, Papez (1937) proposed that “the hypothalamus, the anterior thalamic nuclei, the gyrus cinguli, and the hippocampus, and their interconnections, constitute a harmonious mechanism which may elaborate the functions of central emotion as well as participating in emotional expression.” Although sometimes overlooked by certain theorists, the notion of a “*Papez circuit*” was and continues to be a highly influential concept, in particular because Papez, first, correctly highlighted a major circuit critical for overall hippocampal function; and second, Papez placed the hippocampus clearly within a network connected to a wide variety of anatomically-connected and functionally-conserved structures (Bubb et al., 2017). Papez’ emphasis on emotion was, in certain respects, somewhat misplaced, although ventral hippocampal structures certainly play a role in regulating the HPA axis and, therefore, the stress response (Lowry, 2002; Herman and Mueller, 2006; Ulrich-Lai and Herman, 2009; Myers et al., 2017).

A considerable volume of data from diverse sources supports the idea that the anterior thalamus also plays a critical and central role in explicit, event memory. The phenomenon of diencephalic amnesia (the best known example of which is Korsakoff’s amnesia or syndrome; Albert et al., 1979) strongly suggests that the core mnemonic deficits seen in these patients principally derive from degeneration of the anterior thalamic nuclei, with possible contributions from adjacent midline thalamic nuclei, including the parataenial nucleus (Mair et al., 1979; Mayes et al., 1988; Harding et al., 2000; Segobin et al., 2019). Additionally, studies of patients with rostral thalamic strokes that disrupt the projections from the mammillary bodies to the anterior thalamic nuclei (Carlesimo et al., 2011) add further support for the importance of the anterior thalamic nuclei, along with their mammillary inputs, for episodic memory (Vann and Aggleton, 2004; Vann and Nelson, 2015). Moreover, patients with bilateral fornix damage show deficits in memory on verbal and non-verbal tasks that tax supraspan item memory, while short-term memory tasks are spared (Hodges and Carpenter, 1991; McMackin et al., 1995). Significant correlations between recollective memory and mammillary body volume in such cases further reinforce the notion that hippocampal–medial diencephalic interactions have a vital role in aspects of episodic memory (Tsivilis et al., 2008). Complementary observations have been made in both fornix-transected monkeys (e.g., Gaffan, 1994) and fornix-transected rats (e.g., Ennaceur and Aggleton, 1997; Easton et al., 2009). The key finding across differing patient groups is that the pattern of memory deficits in diencephalic amnesia appears both qualitatively and quantitatively similar to that in amnesics with relatively selective hippocampal pathology (Aggleton, 2008).

## Spatial Coding by Neurons in the Main Hippocampal Output: The Subiculum

The subiculum is the main, yet largely underexplored, output target for hippocampal area CA1 (and, thus, for the hippocampus proper). There have been relatively few investigations of the firing properties of subiculum cells, compared to area CA1 place cells; we briefly survey some here. Unlike area CA1, the subiculum displays a remarkable heterogeneity of spatial responding.

Sharp and Green (1994) found that there were indeed subiculum neurons showing location-specific activity; their firing fields tended to be somewhat larger and more variable than those of area CA1. Subicular cells also displayed ectopic spiking—that is, they showed much higher rates of “out-of-field” firing compared to hippocampal CA1 place cells. They also found that subiculum neurons could be classified as bursting and non-bursting types, based on their spiking, similar to previous observations *in vitro* (see also Stewart and Wong, 1993; Staff et al., 2000; Jung et al., 2001; Anderson and O’Mara, 2003). Recent studies now indicate that sparsely bursting subiculum cells potentially carry more spatial information than other subiculum cell types (Simonnet and Brecht, 2019).

Additionally, Anderson and O’Mara (2004) reported that subiculum cells do not respond to the presence or movement of objects within an arena. Instead, their activity is best predicted by the position and movement of the rat within the arena (behaviorally, the animal did respond to the movement of the objects, suggesting such spatial-object manipulations are subserved by a network independent of the subiculum). Subsequently, Brotons-Mas et al. (2010) reported that about 45% of subiculum place cells show remarkable stability in across multiple light and dark transitions: these cells displayed no evidence for remapping during multiple transitions across light and dark explorations of an open field. The remaining cells showed some degree of remapping, with some units replicating their locational firing across specific light-to-dark conditions, whereas others were strongly affected by light–dark transitions. Brotons-Mas et al. (2010) suggested that because a plurality of units was stable across light–dark transitions, the subiculum participates in or supports the neurocognitive processing underlying path integration because subiculum units appear relatively unresponsive to visual inputs and are perhaps more responsive to cues arising from whole-body movement. Subsequently, Brotons-Mas et al. (2017) conducted extensive spatial phenotyping of subiculum spatial responses using a foraging task in two experimental paradigms—a variably-sized recording arena (small, medium, large), and an arena with systematically inserted barriers in differing locations. Subiculum units showed strongly heterogeneous spatial coding, with place cells, barrier- or perimeter-related cells, as well as boundary-vector cells (Lever et al., 2009), and certain units that showed grid-like patterns of activity in larger arenas.

Adding to this remarkable heterogeneity of cell types, Olson et al. (2017) have described another population of subicular units that code for the axis of travel the animal is currently undertaking. In these experiments, rats foraged in environments with multiple, interconnected paths, of which many had branch-points enabling movement trajectories in opposite directions. Olson et al. (2017) found that about 10% of recorded neurons fired preferentially at head directions approximately 180° apart. Moreover, these firing preferences were preserved during maze rotations, indicating these cells responded to the larger spatial allocentric context, rather than the track itself. These firing preferences were absent during recordings in a trajectory-unconstrained, open-field, circular arena (Olson et al., 2017).

There have been relatively few studies of the subiculum during brain imaging in humans, partly because it is a difficult structure localize using conventional functional magnetic resonance imaging (fMRI) techniques. Two recent studies point to roles for the human subiculum in scene discrimination and head direction processing. Hodgetts et al. (2017), using ultra-high field 7 T high-resolution fMRI, suggest that the subiculum has a particular role during scene, but not face or object, discriminations of previously learned scenes. Kim and Maguire (2018), again using fMRI, had subjects navigate in a 3D space, using a virtual “spaceship.” They found activations in the anterior thalamus and subiculum reflecting the horizontal component of 3D head direction (or “azimuth”), whereas retrosplenial cortex responded to the vertical component of 3D head direction (“pitch”). These data suggest a role for the subiculum in both mnemonic and spatial processing in humans. Meanwhile, a very early study in humans (Vitte et al., 1996), using low-field imaging (1.5 T) suggests that vestibular stimulation (cold irrigation of the external auditory meatus) induced strong activations in the hippocampal formation, subiculum, and retrosplenial cortex (Suzuki et al., 2001, using a similar methodology, also found activations in the human intraparietal sulcus). In addition, a study of whole-body motion in nonhuman primates (O’Mara et al., 1994; Figure 9) reported units responsive to axial and translational movement in the hippocampus proper, as well as in the subiculum. It will be particularly interesting to analyze the nature extent of subicular and thalamic activation in humans with ultrahigh field fMRI resulting from caloric stimulation of the vestibular system.

Tentatively, we can conclude that the subiculum appears to code space in a flexible manner, being involved in the processing of allocentric information, external cues, and path integration, thus broadly supporting spatial navigation. It almost certainly has roles in other forms of memory, given its close connectivity to both the hippocampus proper and the anterior thalamic nuclei, although these roles have not yet been fully explored. An example has been suggested by Deadwyler and Hampson (2004), who found that CA1 and subiculum acted in a complementary fashion to bridge temporal gaps during the performance of a spatial delayed nonmatch-to-sample task.

## Spatial Coding by Neurons in the Anterior Thalamic Nuclei

It has been known for some time that the anterodorsal nucleus of the thalamus contains a substantial population of head-direction cells (Blair and Sharp, 1995; Taube, 1995; Goodridge and Taube, 1997). These cells are thought to contribute to path integration (Frohardt et al., 2006), as well as mapping and navigation in 3D space (Laurens et al., 2016; Page et al., 2018; Angelaki et al., 2019; but see Taube et al., 2013; Shinder and Taube, 2019), the latter function potentially in association with retrosplenial cortex (Kim and Maguire, 2018). Meanwhile, head-direction cells have also been recorded in the anteroventral thalamic nucleus (Tsanov et al., 2011).

Along with the anterodorsal nucleus, the adjacent laterodorsal thalamic nucleus also contains a significant concentration of head-direction cells (Mizumori and Williams, 1993). Again, like the anterodorsal thalamic nucleus, it is reciprocally linked with the retrosplenial cortex, postsubiculum, and presubiculum (van Groen and Wyss, 1992; Clark and Harvey, 2016). Unlike the anterior thalamic nuclei, the laterodorsal nucleus appears to lack inputs from the mammillary bodies, while potentially being more dependent on its visual inputs (Mizumori and Williams, 1993; Clark and Harvey, 2016). Lesions of the rat laterodorsal nucleus cause mild deficits in location learning in the water maze (van Groen et al., 2002) that are exacerbated when the cell loss extends into the adjacent anterodorsal and anteroventral nuclei (Wilton et al., 2001; van Groen et al., 2002). Transient laterodorsal lesions can disrupt hippocampal place cells and impair spatial learning (Mizumori et al., 1994) while neuropathology in this area has been associated with impairments in conscious recollection (Edelstyn et al., 2006). For these reasons, this nucleus appears to parallel the anterodorsal thalamic nucleus, being distinguished by its greater visual inputs.

Returning to the rostral thalamus, much less is known regarding spatial coding in the other anterior thalamic nuclei and other rostral thalamic nuclei, i.e., excluding the anterodorsal nucleus. These additional thalamic sites are, however, of particular interest as the subiculum provides dense, direct inputs to the anteromedial and anteroventral nuclei, contrasting with the projections from the postsubiculum and presubiculum to the anterodorsal thalamic nuclei (Meibach and Siegel, 1977; van Groen and Wyss, 1990a, b; Bubb et al., 2017; Figure 1). Of the various midline nuclei, nucleus reuniens stands out because of its inputs from the subiculum and CA1, as well as its dense, direct projections to the hippocampus, especially to CA1 (Herkenham, 1978; Vertes et al., 2007). Based on their respective connectivity, it had previously been predicted that the anterior thalamic nuclei process information with high spatial and temporal resolution (Aggleton et al., 2010), whereas rostral midline thalamic nuclei have more diffuse roles in attention, control, and arousal (Van der Werf et al., 2002; Vertes et al., 2007). Our current findings strongly support the first prediction but appear to substantially moderate the second prediction, because they show the widespread presence of a diverse population of spatially-responsive neurons in these midline nuclei.

In nucleus reuniens, for example, there is an (unexpected) population of cells coding for head direction (Jankowski et al., 2014), extending the numbers of brain regions coding for head direction to at least 10 (Grieves and Jeffery, 2017). The head-direction cells found in nucleus reuniens are similar in a great many respects to those found in other regions that show head-direction coding: they are not affected by changing visual conditions from light to dark and back to light, or by changing arena shape; they do not remap across days, maintaining a constant orientation. They are also present from the first exposure to the recording environment. Interestingly, theta-cycle skipping cells are also present in nucleus reuniens, similar to the theta-skipping cells found in entorhinal cortex (e.g., Brandon et al., 2013); theta-skipping cells in nucleus reuniens do not code either for head-direction or for place and may instead perform a clock-like or timing function. Finally, nucleus reuniens head-direction cells are not place cells: they are not spatially-modulated in the sense of having a place-related signal (Jankowski et al., 2014).

Nucleus reuniens also provides a substantial anatomical output to hippocampal area CA1, offering a means by which it can modulate spatial coding in the hippocampus proper. For example, combined lesions of the rhomboid nucleus and nucleus reuniens (ReRh) seemingly spare CA1 place cell spatial characteristics in familiar environments, but affect firing in unfamiliar environments (Cholvin et al., 2018). In that experiment, after ReRh lesions, spatial coherence decreased for the first exploration session in a novel environment. Recordings conducted over a 5-day period then showed that ReRh lesions result in a marked and enduring decrease in place field stability and lower firing variability (Cholvin et al., 2018). These data suggest that inputs from ReRh modulate spatial remapping in the hippocampus; it may be that the head directional signal provides a stabilizing directional signal while exploring unfamiliar environments.

Jankowski et al. (2015) recorded in the parataenial nucleus, anteromedial nucleus, and nucleus reuniens, finding place cells and other spatial cells in each of these nuclei. In the parataenial nucleus 29.2% of cells recorded were place cells, whereas 6.2% of cells recorded in the anteromedial nucleus were place cells, with head-direction cells a further 9.7% of recorded cells, with a small number of perimeter/border cells (0.5%). The percentages of place cells recorded in nucleus reuniens was 2.0%, and 2.0% perimeter/border cells; head-direction cells were described separately and quantified at 8.7% (Jankowski et al., 2014). The variation in the percentage of place cells present across these nuclei suggests that the spatial code is more or less sparse for the differing nuclei. More than this, the phenotypic characteristics of the place cells vary in these differing nuclei, with the place fields of cells in nucleus reuniens being the largest and, thereby, carrying the lowest spatial information. Meanwhile, the place fields in the parataenial nucleus occupy the smallest percentage of the recording arena, carrying the highest spatial information. The anteromedial nucleus occupies an intermediate position. In other words, across these anatomically-closely-related nuclei, there appears to be a spatial information content gradient.

In addition to place cells, there is also a population of neurons that respond to the presence of impassable perimeters in the recording arena. These neurons are found in nucleus reuniens and the anteromedial thalamic nucleus (Jankowski et al., 2015). Temporal evolution analyses suggest that the perimeter cells and place cells in these nuclei appear on the first exposure of the animal to the arena. Finally, as noted above, recordings in the anteromedial nucleus of the thalamus also disclose a population of head-direction cells in a hitherto unsuspected and untheorized-about location. These head-direction cells are active also from the first exposure of the animal to the arena, suggesting that early pre-processing of spatial information occurs rapidly in subcortical brain structures.

Perimeters are an important part of the environment, as they formally shape the geometry of the perceptible environment, as well as constraining behavioral trajectories. Perimeters can comprise vertical walls, vertical drops, watercourses, etc.; they can be impassable, or can be passed through at crossings of various types (some perimeters may be perceptual, as in light-to-dark perimeters, perhaps signaling danger or safety). Matulewicz et al. (2019) have surveyed perimeter-related discharge of units in the anteromedial and parataenial nuclei. Matulewicz et al. (2019) found neurons whose firing patterns reflected the presence of walls and drops, irrespective of arena shape (circular or square). Moreover, the firing patterns of these perimeter-responsive units were stable across multiple sleep-wake cycles, and were independent of either light or dark conditions, suggesting that these units are not directly modulated by visual input. Furthermore, these neurons respond in similar ways to both opaque and clear barriers; this latter feature is remarkable because “see-through” or clear barriers (such as Plexiglas) are not part of the natural environment. These neurons respond to perimeter modifications by remapping their firing when two walls of the four present in a rectangular recording arena are removed from recording session to recording session. Further experiments will be required to further explore potential coding differences induced by distal landmarks and proximal tactile cues, under conditions where these information types are explicitly opposed to each other.

## Summary of Spatial Coding by Anterior Thalamic Nuclei

Overall, recordings within the various rostral thalamic nuclei reveal the presence of spatially-responsive cells. The origin of their spatial signals is not yet known, leaving us with (at least) three hypotheses to entertain. The first is that the hippocampal and rostral thalamic spatial systems operate in parallel (in a fashion similar to the accessory optic system); the second is that one is subordinate to the other, for example, the hippocampal formation provides information necessary for the spatial activity observed in the rostral thalamus, or *vice versa*; and third, that there are reciprocal and interdependent relationships between these spatial nodes.

Current findings only provide an incomplete data-set with regard to these three hypotheses. Most of the focus has been on the consequences of anterior thalamic lesions upon hippocampal and parahippocampal activity, stemming from the significance of the anterodorsal nucleus for head-direction signals (Taube, 1995). Consequently, it is known that anterior thalamic lesions cause an absence of postsubiculum head-direction signals (Goodridge and Taube, 1997). While postsubiculum lesions change the properties of anterodorsal head-direction cells, these spatial cells are still present (Goodridge and Taube, 1997), reflecting an asymmetric relationship. Likewise, medial entorhinal cortex lesions leave anterior thalamic head direction activity largely unaffected (Clark and Taube, 2011). In contrast, a disruption of the head-direction network following anterior thalamic lesions impairs parahippocampal “grid cell” activity (Winter et al., 2015). Meanwhile, CA1 place cells appear largely unaffected after anterior thalamic lesions (Calton et al., 2003), although there are alterations to firing patterns in unfamiliar environments after nucleus reuniens lesions (Cholvin et al., 2018). Consequently, with the exception of the head-direction network, little is known about the interdependencies of the various spatial cell types found in the hippocampal formation and anterior thalamus.

## Spatial Signaling and Other Possible Functions Performed by the Claustrum

The claustrum is an underexplored and enigmatic paracortical structure. The claustrum of the rat is a bilateral subcortical sheet of gray matter, spanning approximately the rostral half of the telencephalon, almost to the frontal poles, and caudally to the motor strip. It is bordered by the orbitofrontal cortex, rostrally, both caudally and medially by the caudoputamen, and by the insular cortex laterally (Buchanan and Johnson, 2011; Dillingham et al., 2017, 2019). Anatomically, the claustrum receives projections from the entire cortical mantle. These projections are organized in a topographic fashion, with association cortex projecting to the anterior claustrum and sensory cortices projecting to the posterior portion of the claustrum. In addition, there are also claustrum connections to and from the hippocampal formation, retrosplenial cortex, entorhinal cortex, and a wide variety of subcortical structures, although the existence of projections from the anterior thalamus to the claustrum continues to be a matter of debate (Dillingham et al., 2017, 2019). Comprehensive lesion investigations of the claustrum have yet to be undertaken for technical reasons: the claustrum is a difficult target for the injection-targeting used in lesion analyses, although chemogenetic approaches (such as DREADDs) may prove more tractable.

There have been many hypotheses regarding the functions of the claustrum over the years, most notably that it orchestrates consciousness in the mammalian brain (Crick and Koch, 2005). There have been intermittent anatomical explorations of the claustrum that conclude it does not comprise striatal tissue, but rather can be thought best of as a paracortical tissue. Functional investigations of the claustrum are relatively few in number; we briefly survey some relevant studies here. There have been very few lesion studies of the claustrum. Aclaustral humans are rarely reported: one study of a patient with striking, transient, but symmetric, bilateral, claustral lesions arising from mumps encephalitis (Ishii et al., 2011) reported that during the acute phase of infection the patient experienced visual and auditory hallucinations that resolved with treatment for concurrent epilepsy. With the infection resolved, subsequent imaging showed no persisting lesions of the bilateral claustrum. Intriguingly, Cascella et al. (2011) reported that schizophrenic patients with delusions may have an atrophic left claustrum.

The possible relationship between the claustrum and consciousness continues to receive interest. In an early report, Gabor and Peele (1964) found that electrical stimulation of the claustrum in the awake behaving cat resulted in a state of behavioral relaxation and subsequent induction of sleep.

Koubeissi et al. (2014) investigated a human epilepsy patient implanted with deep-brain electrodes in the claustrum, reporting loss of consciousness on stimulation onset and return of consciousness on stimulus offset. The patient had previously undergone a left hippocampal resection for epilepsy; she was seizure-free for 4 years, after which her seizures returned. Depth electrodes were implanted for seizure sampling. The results of stimulation of the claustral electrode were dramatic: stimulation “resulted in immediate impairment of consciousness, in 10 out of 10 times, with arrest of reading, onset of blank staring, unresponsiveness to auditory or visual commands, and slowing of spontaneous respiratory movements. The patient returned to baseline as soon as the stimulation stopped with no recollection of the events during the stimulation period.” However, a more recent report of bilateral claustral stimulation in five patients (Bickel and Parvizi, 2019) reported that no “changes in subject’s awareness were elicited with unilateral or bilateral electrical perturbation of the claustrum.” It is unclear why the earlier report was not replicated. Perhaps minor differences in electrode placement in the claustrum result in major differences for awareness, or perhaps there is an idiopathic (and perhaps unique) neurological circumstance in the earlier report. Or it might be the case that the claustrum plays a role in orchestrating some of the cortical changes occurring during the wake-sleep transition (Renouard et al., 2015).

Olson and Graybiel (1980) suggested that there is a somatosensory map of the complete surface of the body, arranged longitudinally in the claustrum (in the anesthetized cat preparation). Remedios et al. (2010) have recorded claustral neurons in the conscious, behaving, head-fixed non-human primate, finding neurons responsive to a variety of naturalistic sensory stimuli, especially auditory and visual stimuli. They concluded that the claustrum overall is a multisensory structure, but that individual claustral neurons are unimodal, and that these unimodal neurons are the prevalent neurons present in this preparation. Remedios et al. (2012) also reported in conference proceedings that when aclaustral rats were placed in the center of an eight-arm radial arm maze, they would freeze, apparently unable or unwilling to explore any of the arms. These data await a full report, however.

Jankowski and O’Mara (2015) conducted what appears to be the first freely moving recordings in the rat claustrum. These recordings, remarkably, disclose the existence of a population of spatially-responsive neurons in the claustrum, including place cells, perimeter cells, and object responsive cells. Claustral place cells do not remap during light-dark-light transitions, although their spatial information content falls a little in the dark and is restored in the light. Visual input, therefore, does not seem to affect claustral place cell position, although it does have some effect on claustral place cell spatial information content. Analyses of the temporal evolution of claustral place cells show that they appear to be present from the earliest exposure to the environment. Object responsive cells in the claustrum react to the initial presentation of an object (for example, a glass bottle) with increased firing activity around the object, and near-zero firing activity away from the object. These cells track the repositioning of the object (Jankowski and O’Mara, 2015). Moreover, claustral place cells do not respond to the presence of an object. By contrast, the firing fields of claustral object-responsive cells track the positioning and repositioning of the object in the environment. Claustral units also respond to the perimeter of the environment, showing a penumbra of activity associated with an impassable perimeter. This firing field is very tightly bound to the perimeter, and such cells exhibit near-zero firing rates away from the perimeter itself. Finally, Jankowski and O’Mara (2015) also noted the presence of both theta-modulated and fast-firing bursting cells in the claustrum. The fast-firing cells fired up to 30 Hz or more. These fast-firing cells are not spatially-modulated or spatially-responsive.

One hypothesis that accounts for this unexpected pattern of activity is that the claustrum dynamically represents extended space (in a similar fashion to hippocampal area CA1) but that the claustrum also incorporates landmark (or object) information. We have not, however, observed the presence of head-direction cells in the claustrum. The existence of claustral spatial cells is remarkable, as they are currently unpredicted by any current theory of claustral function, or indeed, any more general theory of the representation of space within the mammalian brain. It may be that the claustrum provides dynamic information about body position and landmark information to the cortex in order to enable moment-to-moment control of behavior.

## Spatial Processing Across Differing Structures

A remarkably diverse, but anatomically interconnected, set of neural structures support similar aspects of spatial coding within the brain. Figure 1 illustrates this contention in respect of anterior thalamic nuclei and related structures. Neurons coding for head-direction are found distributed across eight of these structures; neurons coding for place are found in six of these structures; neurons coding for borders or perimeters are found in four structures; and grid cells are found in at least three of these structures. Notably, several structures code for diverse aspects of space, simultaneously. The subiculum possesses border, grid and place cells, as does nucleus reuniens, for example. The head-direction signal is very widely distributed; in their review, Grieves and Jeffery (2017) find head-directional coding is present in at least eleven distinct but interconnected brain regions (across cortical and subcortical structures). This very widespread distribution of head direction signals suggests head direction coding may play a greater, but more subtle role, in cognition than is generally recognized (O’Mara, 2017). Head direction information may reflect in some structures the continuous recalibration of orienting or attentional responses (e.g., during social interaction; Nummenmaa and Calder, 2009), whereas in other structures it might play a fundamental role in the stable coding of place (see Cholvin et al., 2018). A richer panoply of tasks will be required to test such ideas. The same may be true of the other signals; for many years there was an apparent absence of place cells outside the hippocampus proper. This absences of evidence may simply reflect the conservatism of neural cartographers, rather than a conservatism of the representation of space within diverse brain structures. It is apparent, however, that there is a rich and diverse representation of space far beyond the confines of the entorhinal-hippocampal neuraxis. Moreover, such considerations lend weight to the view that the brain is especially concerned with action-oriented cognitive processing (Gentsch et al., 2016).

## Some Speculations and Conclusions

The hippocampal formation, anterior thalamus, and claustrum show a remarkable rapidity and speed of spatial coding. Maps of space emerge very rapidly and are representationally rich. At present, we do not have a good theoretical account for why there is such redundancy in spatial representations in the brain, nor do we have a good theory to predict across differing neural systems the rapid emergence of such representationally rich coding. One hypothesis worth considering is that of Barsalou (2010) who suggests that “The cognitive system utilizes the environment and the body as external informational structures that complement internal representations….internal representations of a situated character, implemented *via* simulations in the brain’s modal systems, making them well suited for interfacing with external structures”. There has been comparatively little consideration given in contemporary neuroscience to the idea that the brain uses the external environment as a kind of “cognitive surface,” or that it supports the structure of cognition. This might reflect the bias in sensory neuroscience, which has followed the stimulus from the receptor surface into the brain. However, behavioral responses are not aligned along sensory axes; rather, intrinsic activity within the brain determines, in large part, the nature of the processing of sensory stimuli are subjected to (Raichle, 2015; Yamins and DiCarlo, 2016; Dadarlat and Stryker, 2017; Stringer et al., 2019).

The hypothesis that the anterior thalamus engages in extensive pre-processing of stimuli, and generates the elements, or at least some elements of a cognitive map, is an attractive one, for it allows an exploration of the idea that differing types of maps are important at differing temporal scales. Such maps might be adaptively significant, depending on the behavioral and environmental context of the organism. Invoking action maps to escape a predator requires instantaneous action selection and route selection, whereas latent learning during safe periods of exploration might instead operate on an entirely different temporal scale. It may be that the claustrum, given its cortical anatomical position and connectivity allows the rapid selection of action responses in 3D space, when there are temporal constraints requiring the rapid co-ordination of adaptive behavioral responses. The extended hippocampal formation (entorhinal cortex-hippocampus-anterior thalamus) might, by contrast, be more specifically engaged in richer representational action over longer time scales (an idea that finds merit in the possible role of the extended hippocampal formation in imagination and prospection: Buckner, 2010; Maguire and Hassabis, 2011; Zeidman and Maguire, 2016). In turn, it may be that the evolved and adaptive function of memory is to serve present and possible future biological needs, rather than recalling the past in any veridical sense. Hence, the loss of detail over time often observed for many autobiographical experiences; supporting such a view, Conway (2005) suggests, for example, that “for many experiences simply recalling the meaning or the ‘gist’ may be sufficient”.

Finally, we note that biologically-inspired models of spatial navigation rely on core ideas revolving around the dissociation of place information from heading information and their subsequent integration in other downstream areas to facilitate goal-directed navigation (e.g., Barrera and Weitzenfeld, 2008). Here, we suggest that there are important components of this overall theoretical picture missing, in particular relating to an extended and extensive variety of evolutionarily-conserved brain regions (notably the thalamic nuclei, but also the claustrum), which support a wide range of spatial functions. These brain regions continue to be under-explored to this point. We further suggest that significant revision of current models of how the brain controls behavioral choices in a spatial context may be warranted.

## Author Contributions

SO and JA jointly conceived and jointly wrote the manuscript.

## Conflict of Interest Statement

The authors declare that the research was conducted in the absence of any commercial or financial relationships that could be construed as a potential conflict of interest.
